# Possible Role for Bacteriophages in the Treatment of SARS-CoV-2 Infection

**DOI:** 10.1155/2020/8844963

**Published:** 2020-09-19

**Authors:** Vijaya Nath Mishra, Nidhi Kumari, Abhishek Pathak, Rajnish Kumar Chaturvedi, Arun Kumar Gupta, Rameshwar Nath Chaurasia

**Affiliations:** ^1^Department of Neurology, Banaras Hindu University, Varanasi 221005, India; ^2^Developmental Toxicology Laboratory, System Toxicology and Health Risk Assesment Group, Vishvigyan Bhawan, 31 MG Marg, Lucknow, UP 226001, India; ^3^Allahabad High Court, Allahabad 211001, India

## Abstract

An outbreak of severe acute respiratory syndrome coronavirus 2 (SARS-CoV-2) was first reported in Wuhan City, China, in December 2019. Since then, the outbreak has grown into a global pandemic, and neither a vaccine nor a treatment for the disease, termed coronavirus disease 2019 (COVID-19), is currently available. The slow translational progress in the field of research suggests that a large number of studies are urgently required. In this context, this review explores the impact of bacteriophages on SARS-CoV-2, especially concerning phage therapy (PT). Bacteriophages are viruses that infect and kill bacterial cells. Several studies have confirmed that in addition to their antibacterial abilities, bacteriophages also show antiviral and antifungal properties. It has also been shown that PT is effective for building immunity against viral pathogens by reducing the activation of NF kappa B; additionally, phages produce the antiviral protein phagicin. The Ganges river in India, which originates from the Himalayan range, is known to harbor a large number of bacteriophages, which are released into the river gradually by the melting permafrost. Water from this river has traditionally been considered a therapeutic agent for several diseases. In this review, we hypothesize that the Ganges river may play a therapeutic role in the treatment of COVID-19.

## 1. Introduction

The first human case of coronavirus disease 2019 (COVID-19), caused by severe acute respiratory syndrome coronavirus 2 (SARS-CoV-2), was reported by officials in Wuhan City, China, in December 2019. Since then, the outbreak has grown into a global pandemic. As per the United Nations Geoscheme Worldometer'ss COVID-19 data up to 14 July 2020, globally there were 13,360,401 confirmed cases with 580,038 deaths accounting for a mortality rate of approximately 4.3%. In India, the total number of positive cases to date has reached 936,181 with 24,309 deaths (2.6% mortality rate). To minimize the spread of disease and reduce the mortality rate, federal governments have prioritized social distancing and lockdowns as preventive measures. However, if such preventive measures are lifted, the “flattened epidemic curve” tends to start rising gradually again in the absence of any definitive treatment or vaccine. The government of India declared a nationwide lockdown on 25 March 2020 and it was extended up to May 31, 2020.

We now know the detailed structure and sequence of SARS-CoV-2, as well as its pathogenic mechanism in humans. Due to the novel sequence of the virus, there is currently no proven antiviral therapy or vaccine. Studies are ongoing around the globe in order to develop antiviral drugs and a vaccine against SARS-CoV-2.

Progress in studies on bacteriophages has provided new insights into the biology of bacteria and viruses, as well as the positive effects of viruses. Recent evidence also suggests that phages may have therapeutic potential against several diseases, including the seasonal flu and avian influenza [1]. Influenza viruses infect lung tissue similar to SARS-CoV-2. Lauster et al. chemically modified phage capsids which enveloped the influenza virus in such a way that it could no longer infect lung tissue [1]. This phenomenon was studied in a preclinical study using human lung tissue and is being explored against coronavirus infection. Since currently available antiviral drugs attack influenza and coronavirus after they have already infected the lung cells, it is important to target the virus and prevent infection in the first stage of viral infection.

Bacteriophages or phages are viruses that infect and kill bacteria. Bacteriophages consist of a nucleic acid molecule surrounded by a specific protein coat (capsid). The bacteriophage that is found in the River Ganges (or Ganga), especially at its origin, shows the ability to infect several kinds of bacteria. Gomukh is considered the mainspring of the River Ganges. In the opinion of researchers, the Himalayan permafrost traps and holds bacteriophages [2].

In the River Ganga, the proportion of bacteriophages is three times higher than that of bacteria [2]. It has been reported by the National Environmental Engineering Research Institute [3] that the Ganga contains approximately 1,100 types of bacteriophages. This is significantly higher than that in the Yamuna and Narmada, which contain fewer than 200 species of bacteriophages. Ganga water exhibits high alkalinity, and some of its self-purificatory properties contribute to the growth of bacteriophages.

The aforementioned studies encourage further studies on the possibilities of exploring the varied applications of bacteriophages and revival in the frozen Himalayan permafrost.

Phage therapy (PT) was primarily developed to kill bacteria, to help prevent the overuse of antibiotics and the development of antibiotic resistance. Phages mediate immunoregulatory and immunotherapeutic activities that are relevant in balancing the immunological homeostasis of human subjects [4, 5]. Many bacteriophages possess hydrolytic enzymes called lysin, including endolysins and ectolysins, which help to rupture the bacterial peptidoglycan cell wall to allow entry of phage DNA [6]. Moreover, studies have even suggested the efficacy of PT in autoimmune diseases and allergies [7].

PT can also be used against nonbacterial infections like viruses and fungi [8]. Thus, the phages found in the body or Ganga water (phageome) can protect humans from various infections by killing bacteria as well as nonbacterial host-specific organisms [9–11].

It has been found that the quantity of SARS-CoV-2 particles is significantly higher in wastewater [12]. Researchers have suggested that in the case of sewage, a single test is sufficient to determine if the whole population has been infected or not [13]. Coronavirus genetic material (RNA) remains stable as long as it is protected with the capsid, i.e., in the form of a complete virus particle. However, it has been deduced from available information that although the proportion of the virus in sewage water is high, the risk for transmission and infection through this route is very low [14]. Therefore, it is not likely that coronavirus found in sewage water can infect people. This information could be of great importance in managing COVID-19 [15].

This review discusses the possible anti-SARS-CoV-2 effects of bacteriophages from the Ganga River.

## 2. Ganga Water and Phage

Hankin [12] characterized the antibacterial properties of Ganga and Yamuna water long before the concept of bacteriophages was developed by Mallapaty [16]. Hankin reported a cure for diarrhea and cholera by using raw Ganga water. Later, Nautiyal [11] showed the presence of some unknown heat-labile peptides that can kill the pathogenic *Escherichia coli* 0157:H7 [11].

**Figure 1:**
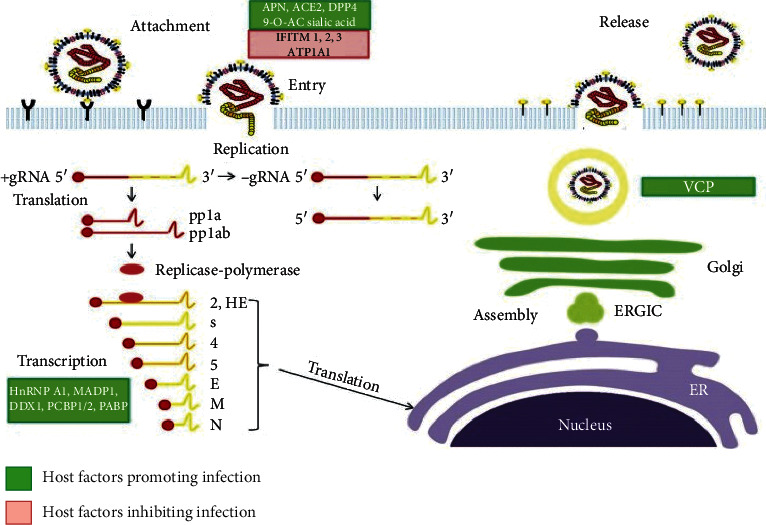
Schematic representation of coronavirus replication cycle (image from Xinyi et al., *Diseases* 4 (3) (2016): 26; https://www.ncbi.nlm.nih.gov/pmc/articles/PMC5456285/).

As shown in Figure 1, coronavirus follows the ligand-receptor binding process and takes over the control over host cytosol following the replication [17]. This further results in the proteolytic cleavage. Phages have antibacterial, antiviral, and antifungal properties [18]. Anti-immunoregulatory and anti-inflammatory activities have also been demonstrated by the phage particles, and these characteristics could be helpful in restoring immunological homeostasis [4]. Thus, phages can offer a protective effect in humans, including in autoimmune diseases and allergies [19].

Repurposing existing therapeutic drugs against new diseases is often a useful strategy for rapid clinical advancement [8]. For example, metformin can reduce the amount of *Bactereoids fragilis* in the intestinal region, which enhances the amount of liver bile and leads to insulin insensitivity. It also exhibits anticancer and enhances lastingness promoting properties [20]. The strategy of repurposing drugs has also emerged as a technique to identify new antiviral agents such as quinine as an antiviral against dengue virus infection [21].

Phagicin, a product obtained from replication of phages, can be detected before morbific phage particles are released from bacterial cells [8]. Phagicin is reactive to trypsin and pepsin, but at the same time shows some resistive properties towards deoxyribonuclease, ribonuclease, and ultraviolet radiation. Thus, phagicin does not infect the host DNA, but it can interfere with viral DNA physiology [22].

Phages can also act as antiviral agents and can significantly reduce the activation of NF kappa B [8]. Bacteriophages have the potential to restrict the replication and absorption of human adenovirus and alter the gene expression of antimicrobial activities [8].

Recent studies have shown that phages have antiviral properties. Bacteriophages are responsible for the production of some antiviral agents which function against harmful viruses. Phagicin, one of the antiviral agents, is the product of phage replication. Phagicin is produced by a phage particle and can be detected in the particle before it is released from the bacterial cell. Phagicin is a protein that interferes with the replication of viral DNA, but it does not cause any harm to the host DNA. Phages in the body compete with the other highly infective eukaryotic viruses for cellular receptors and thereby restrict their harmful actions on the host cell [23].

Phages and phage proteins inhibit the formation of reactive oxygen species (ROS) in response to the result of viral infection. This may explain some of the antiviral activities exhibited by phages [24].

Phages also function to activate natural killer cells (NK cells). This could be an important feature in their therapeutic actions. PT is involved in enhancing immunity after infection. A study of staphylococcal phages on the expression of genes which are involved in antimicrobial immunity in the A549 cell line showed that there is an increased translation of interleukin-2 (IL-2) [25]. IL-2 enhances the activity of NK cells and hence causes a progressive cellular immune response [26].

## 3. Proposed Mechanisms

### 3.1. Phage Therapy and Inactivation of NF-*κ*B during Viral Infection

The phages found in the human body usually transmigrate from the gut and transcytosis in the various tissue and organs like the lungs. On average, around 3 × 10^10^ phages per day get transcytosis in the human body, and this continuous stream of phages is believed to be protective towards antiviral defenses [27]. The expressions of genes that are involved in the immune response are regulated by NF-*κ*B. Viruses have developed a plan of action to utilize NF-*κ*B signaling to replicate and survive within host cells and avoid cellular mechanisms that eliminate the infection [25]. In fact, activation of NF-*κ*B signaling is a prerequisite for some viral infections.

As previously discussed, PT has the potential to build up the active immune response against viral pathogens. Unlike other viruses, phages such as HSV-1 T4 phage do not cause NF-*κ*B activation in human endothelial and epithelial cells. In addition, preincubation of these cells with phages reduced and even terminated the activity of NF-*κ*B [8]. One of the studies confirmed that the staphylococcal phage completely restricts the activation of NF kappa B and its mechanism of action is not related with its antibacterial action [25]. Zhang et al. [25] described in their research that the phage interferes with the HSV induced activation of NF-*κ*B [25]. A review article [28], on the available data, concluded that phages can interfere with the eukaryotic viruses, in vitro and in vivo [29]. It is important to understand the mechanism behind this antiviral functioning of PT.

**Figure 2:**
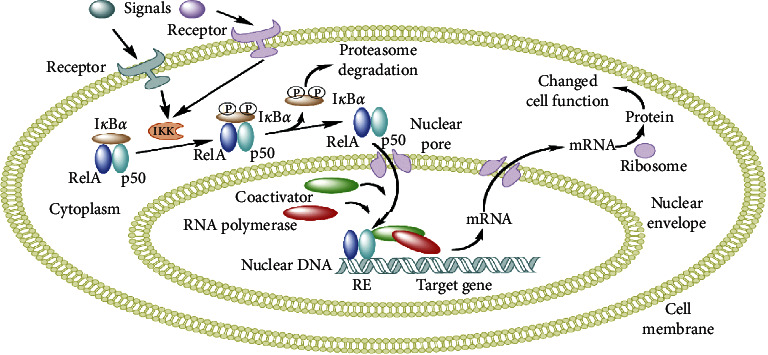
Mechanism of phage action in bacterial cell (Wikimedia Commons. Retrieved 17:38, August 29, 2020, from https://commons.wikimedia.org/w/index.php?title=File:NFKB_mechanism_of_action.png&oldid=232074767).

The NF-*κ*B family consists of seven transcription factors that centrally function in the cellular stress response and inflammation by controlling gene expression [30]. NF-*κ*B also mediates the mechanism of programmed cell death (apoptosis) [31, 32]. NF-*κ*B transcription factors that occur in a dimeric form such as the Rel family have a transactivation domain. In contrast, the homodimeric forms, p50 and p52, are devoid of the transcription activation domain [33]. The action of NF-*κ*B is regulated in a cell type and stimulus-specific manner. As shown in Figure 2, during eukaryotic viral infection, a viral signal binds to a cellular receptor to activate I*κ*B kinase (IKK). This kinase phosphorylates the inhibitor of NF-*κ*B which is I_k_B*α*. Phosphorylated IkB*α* undergoes proteasomal degradation while NF-*κ*B enters the nucleus and binds with other coactivators to activate the gene expression machinery [31].

This mechanism results in the activation of Nk-*κ*B. However, in the presence of phages, this mechanism is hindered due to the phage's protective functioning. Phages downregulate NF-*κ*B activation by blocking phosphorylation of IkB*α* [32]. The NF-*κ*B mechanism is blocked, and the eukaryotic virus is thus unable to activate transcription of the viral genome.

### 3.2. Phage Therapy: Inducer of Anti-Inflammatory Actions

Apart from NF-*κ*B regulation, phages also regulate other processes in a cell for protective functions. A recent study discusses the effects of T4 phage and A5/80 phage on the cellular mechanisms. It concludes that the treatment of the cell with either of the phages leads to the overexpression of the HSPA1 gene. The gene further encodes for the heat shock 70 kDa 1A protein (HSPA1) which is also called Hsp72. Hsp72 is known to perform various cellular activities like protein synthesis, translocation, and folding. Also, when a cell undergoes cellular stress including viral infection, Hsp72 performs cytoprotective function [34].

In a particular study, the experiment demonstrated with the human lung epithelial cells infected with the human adenovirus (Adv) survived after and during the incubation with T4 phage. Preincubation with T4 phage also showed protective activity [35]. It is known that SARS-CoV and SARS-CoV-2 induce apoptosis and result in lymphocytopenia [36, 37]; however, when airway epithelial cells from the human bronchi harvested and cultured phages in vitro, it resulted in reduced apoptosis [38].

The study shows that incubation with A5/80 phage preparation could lead to the TLR10 gene expression [39]. TLR10 is one of the unique genes among the toll-like receptors (TLR) as it functions to prompt the anti-inflammatory effects of a cell during viral infection [39]. A5/80 phage also tends to increase the expression of the interleukin-2 (IL-2) gene. IL-2 propels the activity of natural killer (NK) cells and hence helps the body to perform defense mechanisms against viral infection [34].

Along with TLR10, the TLR2 gene gets activated in response to T4 phage incubation only. TLR2 has a special ability to recognize the common viral coat capsid and consequently promotes the initial antiviral immune response [40].

These data and information regarding phages could possibly help the phage therapy to stand out for the treatment against COVID-19.

## 4. Conclusions and Future Perspective

This review highlights advances in PT. It also summarizes, though very crudely, the important steps in a possible mechanism of using Indian river phages, especially those of the River Ganga, for treatment of the present COVID-19 pandemic. The findings on phages and their possible antiviral properties are preliminary and need to be validated by meticulous in vitro and in vivo studies. If lab studies show some promising results, then it could be possible to have clinical studies and randomized phase 1–3 human trials to prove their therapeutic utility. PT may also hold promise as a treatment for SARS-CoV-2.

## Data Availability

The data used to support the findings of this study are included within the article.
