# Corrigendum to “Possible Role for Bacteriophages in the Treatment of SARS-CoV-2 Infection”

**DOI:** 10.1155/2022/9857582

**Published:** 2022-04-07

**Authors:** Vijaya Nath Mishra, Kumari Nidhi, Abhishek Pathak, Rajnish Kumar Chaturvedi, Arun Kumar Gupta, Rameshwar Nath Chaurasia

**Affiliations:** ^1^Department of Neurology, Banaras Hindu University, Varanasi 221005, India; ^2^Developmental Toxicology Laboratory, System Toxicology and Health Risk Assesment Group, Vishvigyan Bhawan,31 MG Marg, Lucknow, UP 226001, India; ^3^Allahabad High Court, Allahabad 211001, India

In the article titled “Possible Role for Bacteriophages in the Treatment of SARS-CoV-2 Infection” [1], the authors wish to clarify that reference 13 in the article is a study on River Ganga and its self-cleansing properties and not directly related to sewage treatment as suggested. The citation was used in this context because sewage water is entering River Ganga, and the river water was therefore considered to be contaminated with sewage water. Hence, testing the microbes of the water from River Ganga itself would ultimately be helpful in determining if the whole population has been infected with the virus or not.

An additional reference should have been cited, included in the text below, as reference 41 [2]. Accordingly, the following paragraph in the Introduction section should be corrected from “Phage therapy (PT) was primarily developed to kill bacteria, to help prevent the overuse of antibiotics and the development of antibiotic resistance. Phages mediate immunoregulatory and immunotherapeutic activities that are relevant in balancing the immunological homeostasis of human subjects [4, 5]. Many bacteriophages possess hydrolytic enzymes called lysin, including endolysins and ectolysins, which help to rupture the bacterial peptidoglycan cell wall to allow entry of phage DNA [6]. Moreover, studies have even suggested the efficacy of PT in autoimmune diseases and allergies [7]” to “Phage therapy (PT) was primarily developed to kill bacteria, to help prevent the overuse of antibiotics and the development of antibiotic resistance. Phages mediate immunoregulatory and immunotherapeutic activities that are relevant in balancing the immunological homeostasis of human subjects [4, 5]. Many bacteriophages possess hydrolytic enzymes called lysin, including endolysins and ectolysins, which help to rupture the bacterial peptidoglycan cell wall to allow entry of phage DNA [6]. Moreover, studies have even suggested the efficacy of PT in autoimmune diseases and allergies [7]. One of the studies also concludes that during the viral infection, some of the bacterial colony also tends to spread vigorously in an infected human body. Like the infection of *Acinetobacter baumanii* and *Klebsiella pneumoniae* bacteria have been found in the COVID-19 patients. This kind of infection can also cause sepsis, and in that situation, phages have been found helpful to treat the bacterial infection also in the COVID-19 patients [41].”
